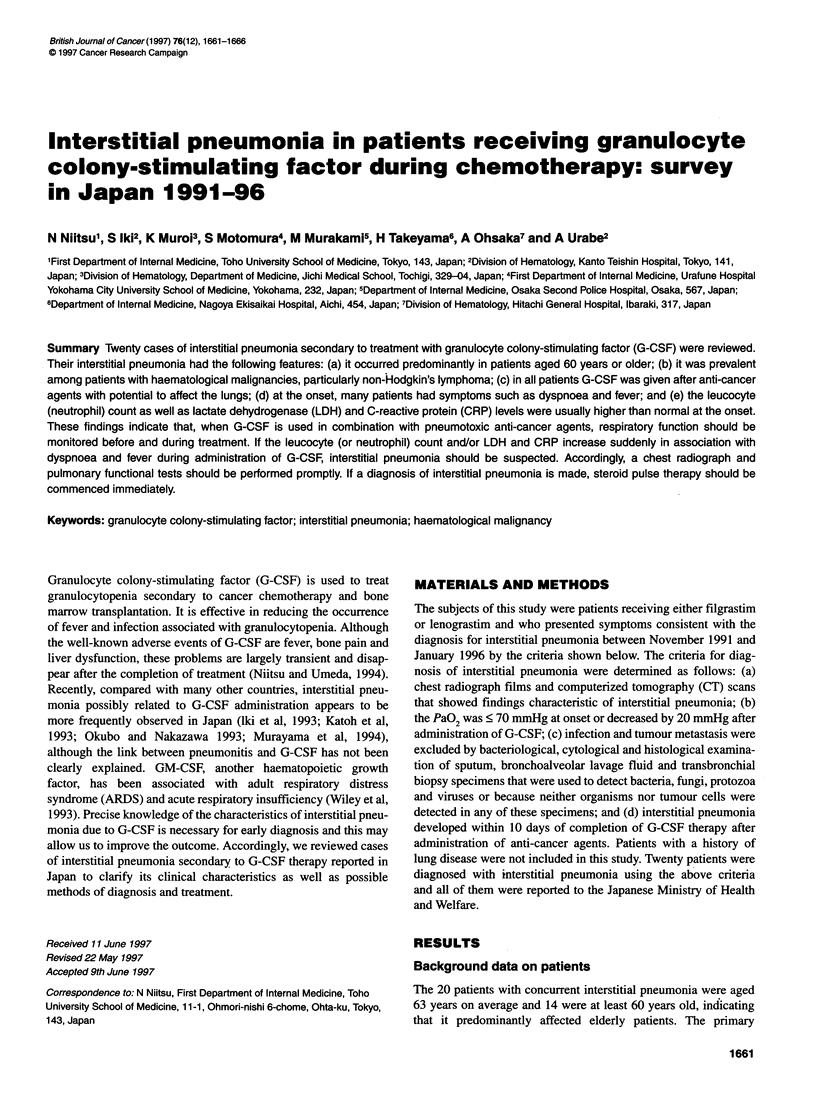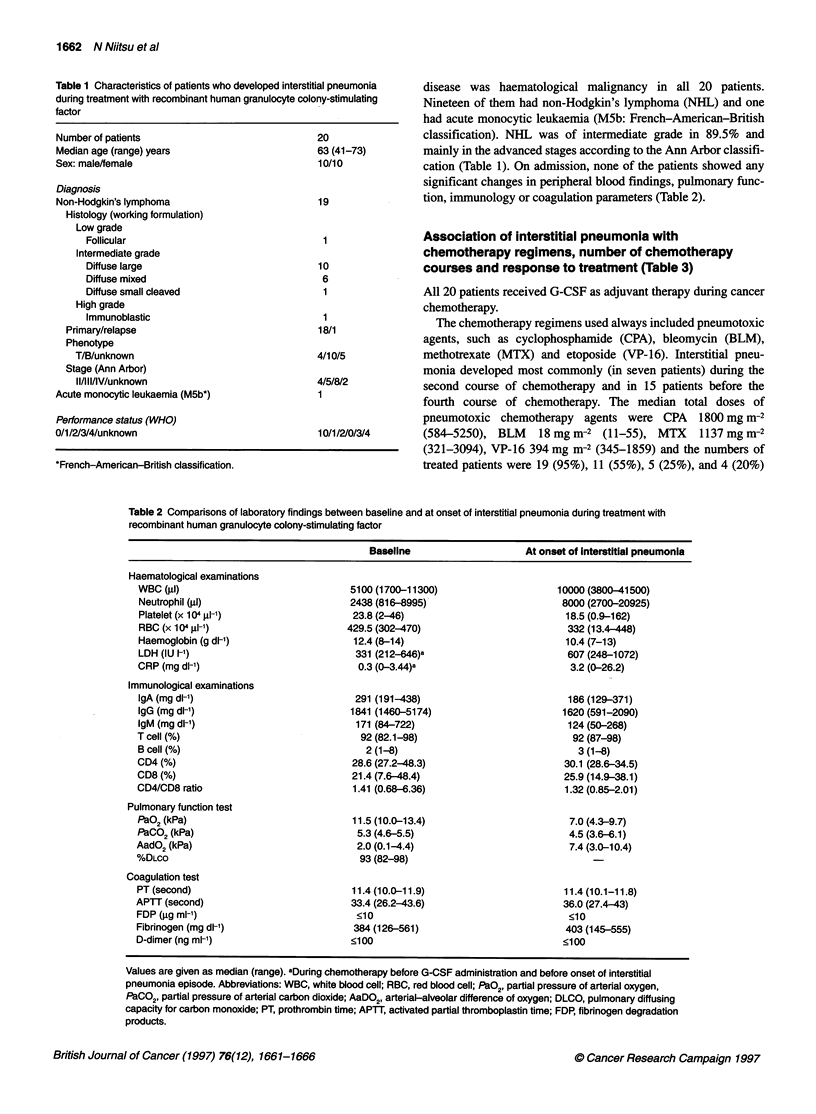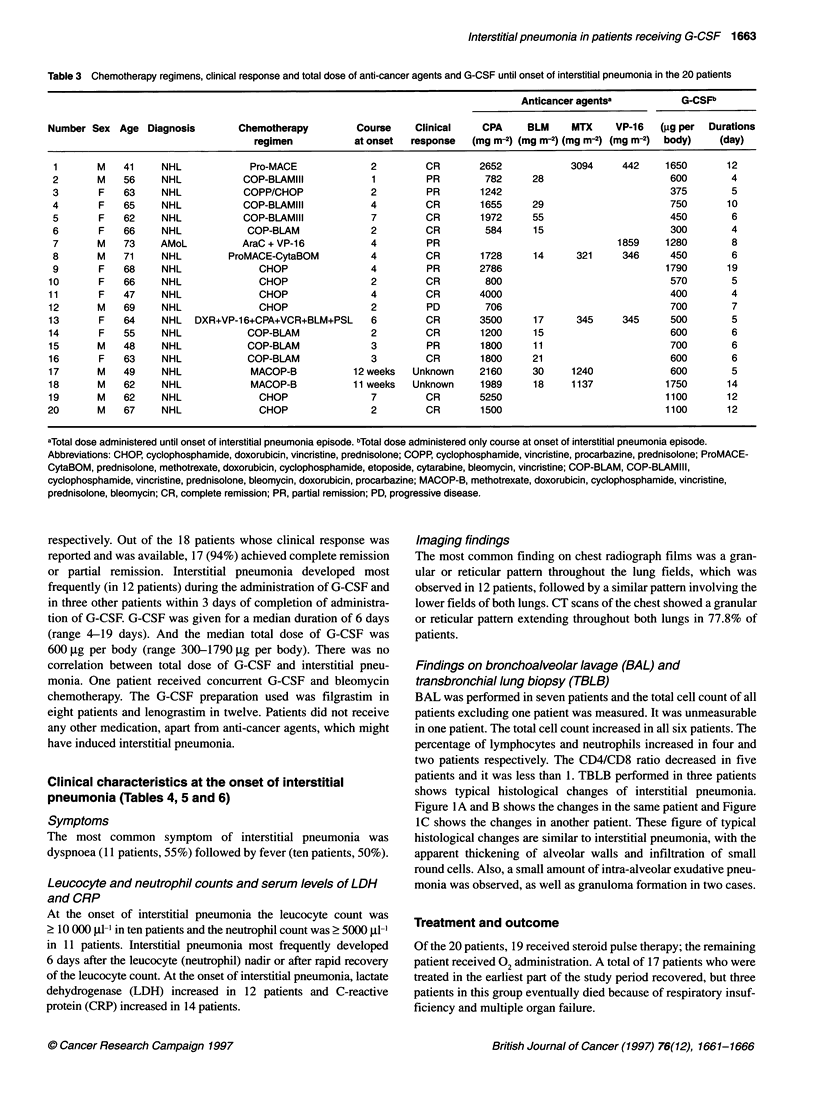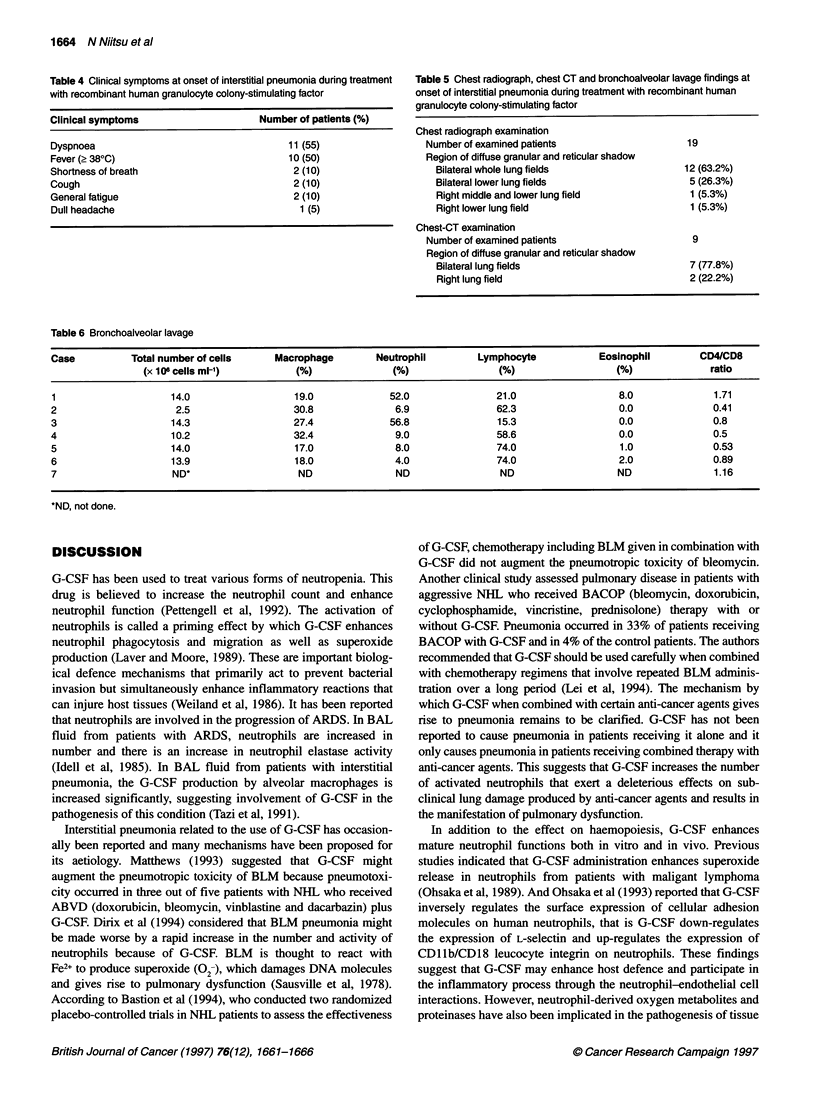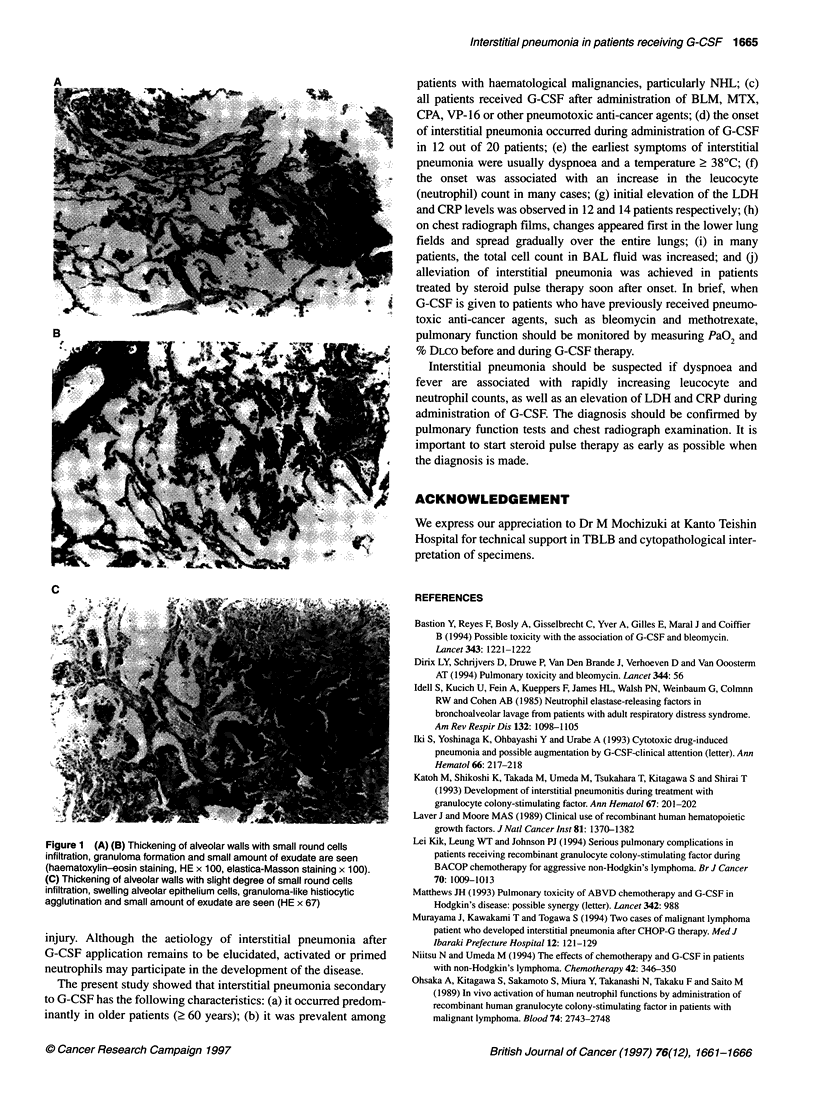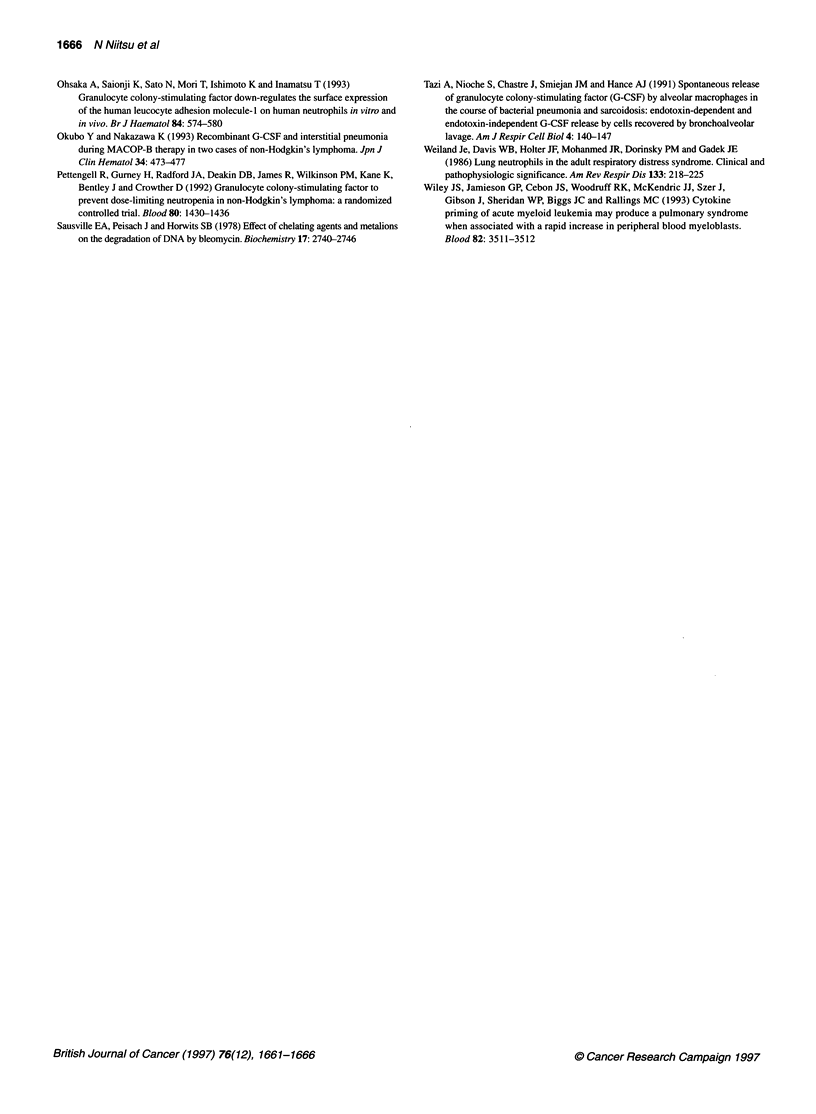# Interstitial pneumonia in patients receiving granulocyte colony-stimulating factor during chemotherapy: survey in Japan 1991-96.

**DOI:** 10.1038/bjc.1997.614

**Published:** 1997

**Authors:** N. Niitsu, S. Iki, K. Muroi, S. Motomura, M. Murakami, H. Takeyama, A. Ohsaka, A. Urabe

**Affiliations:** First Department of Internal Medicine, Toho University School of Medicine, Tokyo, Japan.

## Abstract

**Images:**


British Journal of Cancer (1997) 76(12), 1661–1666
© 1997 Cancer Research Campaign

### Interstitial pneumonia in patients receiving granulocyte colony-stimulating factor during chemotherapy: survey in Japan 1991–96

**N Niitsu[1], S Iki[2], K Muroi[3], S Motomura[4], M Murakami[5], H Takeyama[6], A Ohsaka[7] and A Urabe[2]**

[1]First Department of Internal Medicine, Toho University School of Medicine, Tokyo, 143, Japan; [2]Division of Hematology, Kanto Teishin Hospital, Tokyo, 141, Japan; [3]Division of Hematology, Department of Medicine, Jichi Medical School, Tochigi, 329–04, Japan; [4]First Department of Internal Medicine, Urafune Hospital Yokohama City University School of Medicine, Yokohama, 232, Japan; [5]Department of Internal Medicine, Osaka Second Police Hospital, Osaka, 567, Japan; [6]Department of Internal Medicine, Nagoya Ekisaikai Hospital, Aichi, 454, Japan; [7]Division of Hematology, Hitachi General Hospital, Ibaraki, 317, Japan

**Summary** Twenty cases of interstitial pneumonia secondary to treatment with granulocyte colony-stimulating factor (G-CSF) were reviewed. Their interstitial pneumonia had the following features: (a) it occurred predominantly in patients aged 60 years or older; (b) it was prevalent among patients with haematological malignancies, particularly non-Hodgkin's lymphoma; (c) in all patients G-CSF was given after anti-cancer agents with potential to affect the lungs; (d) at the onset, many patients had symptoms such as dyspnoea and fever; and (e) the leucocyte (neutrophil) count as well as lactate dehydrogenase (LDH) and C-reactive protein (CRP) levels were usually higher than normal at the onset. These findings indicate that, when G-CSF is used in combination with pneumotoxic anti-cancer agents, respiratory function should be monitored before and during treatment. If the leucocyte (or neutrophil) count and/or LDH and CRP increase suddenly in association with dyspnoea and fever during administration of G-CSF, interstitial pneumonia should be suspected. Accordingly, a chest radiograph and pulmonary functional tests should be performed promptly. If a diagnosis of interstitial pneumonia is made, steroid pulse therapy should be commenced immediately.

**Keywords:** granulocyte colony-stimulating factor; interstitial pneumonia; haematological malignancy

Granulocyte colony-stimulating factor (G-CSF) is used to treat granulocytopenia secondary to cancer chemotherapy and bone marrow transplantation. It is effective in reducing the occurrence of fever and infection associated with granulocytopenia. Although the well-known adverse events of G-CSF are fever, bone pain and liver dysfunction, these problems are largely transient and disappear after the completion of treatment (Niitsu and Umeda, 1994). Recently, compared with many other countries, interstitial pneumonia possibly related to G-CSF administration appears to be more frequently observed in Japan (Iki et al, 1993; Katoh et al, 1993; Okubo and Nakazawa 1993; Murayama et al, 1994), although the link between pneumonitis and G-CSF has not been clearly explained. GM-CSF, another haematopoietic growth factor, has been associated with adult respiratory distress syndrome (ARDS) and acute respiratory insufficiency (Wiley et al, 1993). Precise knowledge of the characteristics of interstitial pneumonia due to G-CSF is necessary for early diagnosis and this may allow us to improve the outcome. Accordingly, we reviewed cases of interstitial pneumonia secondary to G-CSF therapy reported in Japan to clarify its clinical characteristics as well as possible methods of diagnosis and treatment.

*Received 11 June 1997*
*Revised 22 May 1997*
*Accepted 9th June 1997*

*Correspondence to:* N Niitsu, First Department of Internal Medicine, Toho University School of Medicine, 11-1, Ohmori-nishi 6-chome, Ohta-ku, Tokyo, 143, Japan

### MATERIALS AND METHODS

The subjects of this study were patients receiving either filgrastim or lenograstim and who presented symptoms consistent with the diagnosis for interstitial pneumonia between November 1991 and January 1996 by the criteria shown below. The criteria for diagnosis of interstitial pneumonia were determined as follows: (a) chest radiograph films and computerized tomography (CT) scans that showed findings characteristic of interstitial pneumonia; (b) the $PaO_2$ was $\leq$ 70 mmHg at onset or decreased by 20 mmHg after administration of G-CSF; (c) infection and tumour metastasis were excluded by bacteriological, cytological and histological examination of sputum, bronchoalveolar lavage fluid and transbronchial biopsy specimens that were used to detect bacteria, fungi, protozoa and viruses or because neither organisms nor tumour cells were detected in any of these specimens; and (d) interstitial pneumonia developed within 10 days of completion of G-CSF therapy after administration of anti-cancer agents. Patients with a history of lung disease were not included in this study. Twenty patients were diagnosed with interstitial pneumonia using the above criteria and all of them were reported to the Japanese Ministry of Health and Welfare.

### RESULTS

### Background data on patients

The 20 patients with concurrent interstitial pneumonia were aged 63 years on average and 14 were at least 60 years old, indicating that it predominantly affected elderly patients. The primary

**Table 1** Characteristics of patients who developed interstitial pneumonia during treatment with recombinant human granulocyte colony-stimulating factor

| | |
|---|---|
| Number of patients | 20 |
| Median age (range) years | 63 (41–73) |
| Sex: male/female | 10/10 |
| *Diagnosis* | |
| Non-Hodgkin's lymphoma | 19 |
| Histology (working formulation) | |
| Low grade | |
| Follicular | 1 |
| Intermediate grade | |
| Diffuse large | 10 |
| Diffuse mixed | 6 |
| Diffuse small cleaved | 1 |
| High grade | |
| Immunoblastic | 1 |
| Primary/relapse | 18/1 |
| Phenotype | |
| T/B/unknown | 4/10/5 |
| Stage (Ann Arbor) | |
| II/III/IV/unknown | 4/5/8/2 |
| Acute monocytic leukaemia (M5b*) | 1 |
| *Performance status (WHO)* | |
| 0/1/2/3/4/unknown | 10/1/2/0/3/4 |

*French–American–British classification.

disease was haematological malignancy in all 20 patients. Nineteen of them had non-Hodgkin's lymphoma (NHL) and one had acute monocytic leukaemia (M5b: French–American–British classification). NHL was of intermediate grade in 89.5% and mainly in the advanced stages according to the Ann Arbor classification (Table 1). On admission, none of the patients showed any significant changes in peripheral blood findings, pulmonary function, immunology or coagulation parameters (Table 2).

### Association of interstitial pneumonia with chemotherapy regimens, number of chemotherapy courses and response to treatment (Table 3)

All 20 patients received G-CSF as adjuvant therapy during cancer chemotherapy.

The chemotherapy regimens used always included pneumotoxic agents, such as cyclophosphamide (CPA), bleomycin (BLM), methotrexate (MTX) and etoposide (VP-16). Interstitial pneumonia developed most commonly (in seven patients) during the second course of chemotherapy and in 15 patients before the fourth course of chemotherapy. The median total doses of pneumotoxic chemotherapy agents were CPA 1800 mg $m^{-2}$ (584–5250), BLM 18 mg $m^{-2}$ (11–55), MTX 1137 mg $m^{-2}$ (321–3094), VP-16 394 mg $m^{-2}$ (345–1859) and the numbers of treated patients were 19 (95%), 11 (55%), 5 (25%), and 4 (20%)

**Table 2** Comparisons of laboratory findings between baseline and at onset of interstitial pneumonia during treatment with recombinant human granulocyte colony-stimulating factor

| | Baseline | At onset of interstitial pneumonia |
|---|---|---|
| Haematological examinations | | |
| WBC (μl) | 5100 (1700–11300) | 10000 (3800–41500) |
| Neutrophil (μl) | 2438 (816–8995) | 8000 (2700–20925) |
| Platelet (× $10^4$ $\mu l^{-1}$) | 23.8 (2–46) | 18.5 (0.9–162) |
| RBC (× $10^4$ $\mu l^{-1}$) | 429.5 (302–470) | 332 (13.4–448) |
| Haemoglobin (g $dl^{-1}$) | 12.4 (8–14) | 10.4 (7–13) |
| LDH (IU $l^{-1}$) | 331 (212–646)[a] | 607 (248–1072) |
| CRP (mg $dl^{-1}$) | 0.3 (0–3.44)[a] | 3.2 (0–26.2) |
| Immunological examinations | | |
| IgA (mg $dl^{-1}$) | 291 (191–438) | 186 (129–371) |
| IgG (mg $dl^{-1}$) | 1841 (1460–5174) | 1620 (591–2090) |
| IgM (mg $dl^{-1}$) | 171 (84–722) | 124 (50–268) |
| T cell (%) | 92 (82.1–98) | 92 (87–98) |
| B cell (%) | 2 (1–8) | 3 (1–8) |
| CD4 (%) | 28.6 (27.2–48.3) | 30.1 (28.6–34.5) |
| CD8 (%) | 21.4 (7.6–48.4) | 25.9 (14.9–38.1) |
| CD4/CD8 ratio | 1.41 (0.68–6.36) | 1.32 (0.85–2.01) |
| Pulmonary function test | | |
| $PaO_2$ (kPa) | 11.5 (10.0–13.4) | 7.0 (4.3–9.7) |
| $PaCO_2$ (kPa) | 5.3 (4.6–5.5) | 4.5 (3.6–6.1) |
| $AadO_2$ (kPa) | 2.0 (0.1–4.4) | 7.4 (3.0–10.4) |
| %DLCO | 93 (82–98) | — |
| Coagulation test | | |
| PT (second) | 11.4 (10.0–11.9) | 11.4 (10.1–11.8) |
| APTT (second) | 33.4 (26.2–43.6) | 36.0 (27.4–43) |
| FDP (μg $ml^{-1}$) | ≤10 | ≤10 |
| Fibrinogen (mg $dl^{-1}$) | 384 (126–561) | 403 (145–555) |
| D-dimer (ng $ml^{-1}$) | ≤100 | ≤100 |

Values are given as median (range). [a]During chemotherapy before G-CSF administration and before onset of interstitial pneumonia episode. Abbreviations: WBC, white blood cell; RBC, red blood cell; $PaO_2$, partial pressure of arterial oxygen, $PaCO_2$, partial pressure of arterial carbon dioxide; $AaDO_2$, arterial–alveolar difference of oxygen; DLCO, pulmonary diffusing capacity for carbon monoxide; PT, prothrombin time; APTT, activated partial thromboplastin time; FDP, fibrinogen degradation products.

*© Cancer Research Campaign 1997*

**Table 3** Chemotherapy regimens, clinical response and total dose of anti-cancer agents and G-CSF until onset of interstitial pneumonia in the 20 patients

| | | | | | | | Anticancer agents[a] | | | | G-CSF[b] | |
|---|---|---|---|---|---|---|---|---|---|---|---|---|
| Number | Sex | Age | Diagnosis | Chemotherapy regimen | Course at onset | Clinical response | CPA (mg $m^{-2}$) | BLM (mg $m^{-2}$) | MTX (mg $m^{-2}$) | VP-16 (mg $m^{-2}$) | (µg per body) | Durations (day) |
| 1 | M | 41 | NHL | Pro-MACE | 2 | CR | 2652 | | 3094 | 442 | 1650 | 12 |
| 2 | M | 56 | NHL | COP-BLAMIII | 1 | PR | 782 | 28 | | | 600 | 4 |
| 3 | F | 63 | NHL | COPP/CHOP | 2 | PR | 1242 | | | | 375 | 5 |
| 4 | F | 65 | NHL | COP-BLAMIII | 4 | CR | 1655 | 29 | | | 750 | 10 |
| 5 | F | 62 | NHL | COP-BLAMIII | 7 | CR | 1972 | 55 | | | 450 | 6 |
| 6 | F | 66 | NHL | COP-BLAM | 2 | CR | 584 | 15 | | | 300 | 4 |
| 7 | M | 73 | AMoL | AraC + VP-16 | 4 | PR | | | | 1859 | 1280 | 8 |
| 8 | M | 71 | NHL | ProMACE-CytaBOM | 4 | CR | 1728 | 14 | 321 | 346 | 450 | 6 |
| 9 | F | 68 | NHL | CHOP | 4 | PR | 2786 | | | | 1790 | 19 |
| 10 | F | 66 | NHL | CHOP | 2 | CR | 800 | | | | 570 | 5 |
| 11 | F | 47 | NHL | CHOP | 4 | CR | 4000 | | | | 400 | 4 |
| 12 | M | 69 | NHL | CHOP | 2 | PD | 706 | | | | 700 | 7 |
| 13 | F | 64 | NHL | DXR+VP-16+CPA+VCR+BLM+PSL | 6 | CR | 3500 | 17 | 345 | 345 | 500 | 5 |
| 14 | F | 55 | NHL | COP-BLAM | 2 | CR | 1200 | 15 | | | 600 | 6 |
| 15 | M | 48 | NHL | COP-BLAM | 3 | PR | 1800 | 11 | | | 700 | 6 |
| 16 | F | 63 | NHL | COP-BLAM | 3 | CR | 1800 | 21 | | | 600 | 6 |
| 17 | M | 49 | NHL | MACOP-B | 12 weeks | Unknown | 2160 | 30 | 1240 | | 600 | 5 |
| 18 | M | 62 | NHL | MACOP-B | 11 weeks | Unknown | 1989 | 18 | 1137 | | 1750 | 14 |
| 19 | M | 62 | NHL | CHOP | 7 | CR | 5250 | | | | 1100 | 12 |
| 20 | M | 67 | NHL | CHOP | 2 | CR | 1500 | | | | 1100 | 12 |

[a]Total dose administered until onset of interstitial pneumonia episode. [b]Total dose administered only course at onset of interstitial pneumonia episode. Abbreviations: CHOP, cyclophosphamide, doxorubicin, vincristine, prednisolone; COPP, cyclophosphamide, vincristine, procarbazine, prednisolone; ProMACE-CytaBOM, prednisolone, methotrexate, doxorubicin, cyclophosphamide, etoposide, cytarabine, bleomycin, vincristine; COP-BLAM, COP-BLAMIII, cyclophosphamide, vincristine, prednisolone, bleomycin, doxorubicin, procarbazine; MACOP-B, methotrexate, doxorubicin, cyclophosphamide, vincristine, prednisolone, bleomycin; CR, complete remission; PR, partial remission; PD, progressive disease.

respectively. Out of the 18 patients whose clinical response was reported and was available, 17 (94%) achieved complete remission or partial remission. Interstitial pneumonia developed most frequently (in 12 patients) during the administration of G-CSF and in three other patients within 3 days of completion of administration of G-CSF. G-CSF was given for a median duration of 6 days (range 4–19 days). And the median total dose of G-CSF was 600 µg per body (range 300–1790 µg per body). There was no correlation between total dose of G-CSF and interstitial pneumonia. One patient received concurrent G-CSF and bleomycin chemotherapy. The G-CSF preparation used was filgrastim in eight patients and lenograstim in twelve. Patients did not receive any other medication, apart from anti-cancer agents, which might have induced interstitial pneumonia.

### Clinical characteristics at the onset of interstitial pneumonia (Tables 4, 5 and 6)

### *Symptoms*

The most common symptom of interstitial pneumonia was dyspnoea (11 patients, 55%) followed by fever (ten patients, 50%).

### *Leucocyte and neutrophil counts and serum levels of LDH and CRP*

At the onset of interstitial pneumonia the leucocyte count was $\geq$ 10 000 $\mu l^{-1}$ in ten patients and the neutrophil count was $\geq$ 5000 $\mu l^{-1}$ in 11 patients. Interstitial pneumonia most frequently developed 6 days after the leucocyte (neutrophil) nadir or after rapid recovery of the leucocyte count. At the onset of interstitial pneumonia, lactate dehydrogenase (LDH) increased in 12 patients and C-reactive protein (CRP) increased in 14 patients.

### *Imaging findings*

The most common finding on chest radiograph films was a granular or reticular pattern throughout the lung fields, which was observed in 12 patients, followed by a similar pattern involving the lower fields of both lungs. CT scans of the chest showed a granular or reticular pattern extending throughout both lungs in 77.8% of patients.

### *Findings on bronchoalveolar lavage (BAL) and transbronchial lung biopsy (TBLB)*

BAL was performed in seven patients and the total cell count of all patients excluding one patient was measured. It was unmeasurable in one patient. The total cell count increased in all six patients. The percentage of lymphocytes and neutrophils increased in four and two patients respectively. The CD4/CD8 ratio decreased in five patients and it was less than 1. TBLB performed in three patients shows typical histological changes of interstitial pneumonia. Figure 1A and B shows the changes in the same patient and Figure 1C shows the changes in another patient. These figure of typical histological changes are similar to interstitial pneumonia, with the apparent thickening of alveolar walls and infiltration of small round cells. Also, a small amount of intra-alveolar exudative pneumonia was observed, as well as granuloma formation in two cases.

### Treatment and outcome

Of the 20 patients, 19 received steroid pulse therapy; the remaining patient received $O_2$ administration. A total of 17 patients who were treated in the earliest part of the study period recovered, but three patients in this group eventually died because of respiratory insufficiency and multiple organ failure.

**Table 4** Clinical symptoms at onset of interstitial pneumonia during treatment with recombinant human granulocyte colony-stimulating factor

| Clinical symptoms | Number of patients (%) |
|---|---|
| Dyspnoea | 11 (55) |
| Fever (≥ 38°C) | 10 (50) |
| Shortness of breath | 2 (10) |
| Cough | 2 (10) |
| General fatigue | 2 (10) |
| Dull headache | 1 (5) |

**Table 5** Chest radiograph, chest CT and bronchoalveolar lavage findings at onset of interstitial pneumonia during treatment with recombinant human granulocyte colony-stimulating factor

| | |
|---|---|
| Chest radiograph examination | |
| Number of examined patients | 19 |
| Region of diffuse granular and reticular shadow | |
| Bilateral whole lung fields | 12 (63.2%) |
| Bilateral lower lung fields | 5 (26.3%) |
| Right middle and lower lung field | 1 (5.3%) |
| Right lower lung field | 1 (5.3%) |
| Chest-CT examination | |
| Number of examined patients | 9 |
| Region of diffuse granular and reticular shadow | |
| Bilateral lung fields | 7 (77.8%) |
| Right lung field | 2 (22.2%) |

**Table 6** Bronchoalveolar lavage

| Case | Total number of cells ($\times 10^6$ cells $ml^{-1}$) | Macrophage (%) | Neutrophil (%) | Lymphocyte (%) | Eosinophil (%) | CD4/CD8 ratio |
|---|---|---|---|---|---|---|
| 1 | 14.0 | 19.0 | 52.0 | 21.0 | 8.0 | 1.71 |
| 2 | 2.5 | 30.8 | 6.9 | 62.3 | 0.0 | 0.41 |
| 3 | 14.3 | 27.4 | 56.8 | 15.3 | 0.0 | 0.8 |
| 4 | 10.2 | 32.4 | 9.0 | 58.6 | 0.0 | 0.5 |
| 5 | 14.0 | 17.0 | 8.0 | 74.0 | 1.0 | 0.53 |
| 6 | 13.9 | 18.0 | 4.0 | 74.0 | 2.0 | 0.89 |
| 7 | ND* | ND | ND | ND | ND | 1.16 |

*ND, not done.

### DISCUSSION

G-CSF has been used to treat various forms of neutropenia. This drug is believed to increase the neutrophil count and enhance neutrophil function (Pettengell et al, 1992). The activation of neutrophils is called a priming effect by which G-CSF enhances neutrophil phagocytosis and migration as well as superoxide production (Laver and Moore, 1989). These are important biological defence mechanisms that primarily act to prevent bacterial invasion but simultaneously enhance inflammatory reactions that can injure host tissues (Weiland et al, 1986). It has been reported that neutrophils are involved in the progression of ARDS. In BAL fluid from patients with ARDS, neutrophils are increased in number and there is an increase in neutrophil elastase activity (Idell et al, 1985). In BAL fluid from patients with interstitial pneumonia, the G-CSF production by alveolar macrophages is increased significantly, suggesting involvement of G-CSF in the pathogenesis of this condition (Tazi et al, 1991).

Interstitial pneumonia related to the use of G-CSF has occasionally been reported and many mechanisms have been proposed for its aetiology. Matthews (1993) suggested that G-CSF might augment the pneumotropic toxicity of BLM because pneumotoxicity occurred in three out of five patients with NHL who received ABVD (doxorubicin, bleomycin, vinblastine and dacarbazin) plus G-CSF. Dirix et al (1994) considered that BLM pneumonia might be made worse by a rapid increase in the number and activity of neutrophils because of G-CSF. BLM is thought to react with $Fe^{2+}$ to produce superoxide ($O_2^-$), which damages DNA molecules and gives rise to pulmonary dysfunction (Sausville et al, 1978). According to Bastion et al (1994), who conducted two randomized placebo-controlled trials in NHL patients to assess the effectiveness of G-CSF, chemotherapy including BLM given in combination with G-CSF did not augment the pneumotropic toxicity of bleomycin. Another clinical study assessed pulmonary disease in patients with aggressive NHL who received BACOP (bleomycin, doxorubicin, cyclophosphamide, vincristine, prednisolone) therapy with or without G-CSF. Pneumonia occurred in 33% of patients receiving BACOP with G-CSF and in 4% of the control patients. The authors recommended that G-CSF should be used carefully when combined with chemotherapy regimens that involve repeated BLM administration over a long period (Lei et al, 1994). The mechanism by which G-CSF when combined with certain anti-cancer agents gives rise to pneumonia remains to be clarified. G-CSF has not been reported to cause pneumonia in patients receiving it alone and it only causes pneumonia in patients receiving combined therapy with anti-cancer agents. This suggests that G-CSF increases the number of activated neutrophils that exert a deleterious effects on subclinical lung damage produced by anti-cancer agents and results in the manifestation of pulmonary dysfunction.

In addition to the effect on haemopoiesis, G-CSF enhances mature neutrophil functions both in vitro and in vivo. Previous studies indicated that G-CSF administration enhances superoxide release in neutrophils from patients with maligant lymphoma (Ohsaka et al, 1989). And Ohsaka et al (1993) reported that G-CSF inversely regulates the surface expression of cellular adhesion molecules on human neutrophils, that is G-CSF down-regulates the expression of L-selectin and up-regulates the expression of CD11b/CD18 leucocyte integrin on neutrophils. These findings suggest that G-CSF may enhance host defence and participate in the inflammatory process through the neutrophil–endothelial cell interactions. However, neutrophil-derived oxygen metabolites and proteinases have also been implicated in the pathogenesis of tissue

© Cancer Research Campaign 1997

**Figure 1** **(A) (B)** Thickening of alveolar walls with small round cells infiltration, granuloma formation and small amount of exudate are seen (haematoxylin–eosin staining, HE × 100, elastica-Masson staining × 100). **(C)** Thickening of alveolar walls with slight degree of small round cells infiltration, swelling alveolar epithelium cells, granuloma-like histiocytic agglutination and small amount of exudate are seen (HE × 67)

injury. Although the aetiology of interstitial pneumonia after G-CSF application remains to be elucidated, activated or primed neutrophils may participate in the development of the disease.

The present study showed that interstitial pneumonia secondary to G-CSF has the following characteristics: (a) it occurred predominantly in older patients (≥ 60 years); (b) it was prevalent among patients with haematological malignancies, particularly NHL; (c) all patients received G-CSF after administration of BLM, MTX, CPA, VP-16 or other pneumotoxic anti-cancer agents; (d) the onset of interstitial pneumonia occurred during administration of G-CSF in 12 out of 20 patients; (e) the earliest symptoms of interstitial pneumonia were usually dyspnoea and a temperature ≥ 38°C; (f) the onset was associated with an increase in the leucocyte (neutrophil) count in many cases; (g) initial elevation of the LDH and CRP levels was observed in 12 and 14 patients respectively; (h) on chest radiograph films, changes appeared first in the lower lung fields and spread gradually over the entire lungs; (i) in many patients, the total cell count in BAL fluid was increased; and (j) alleviation of interstitial pneumonia was achieved in patients treated by steroid pulse therapy soon after onset. In brief, when G-CSF is given to patients who have previously received pneumotoxic anti-cancer agents, such as bleomycin and methotrexate, pulmonary function should be monitored by measuring $Pa O_2$ and % DLCO before and during G-CSF therapy.

Interstitial pneumonia should be suspected if dyspnoea and fever are associated with rapidly increasing leucocyte and neutrophil counts, as well as an elevation of LDH and CRP during administration of G-CSF. The diagnosis should be confirmed by pulmonary function tests and chest radiograph examination. It is important to start steroid pulse therapy as early as possible when the diagnosis is made.

### ACKNOWLEDGEMENT

We express our appreciation to Dr M Mochizuki at Kanto Teishin Hospital for technical support in TBLB and cytopathological interpretation of specimens.

### REFERENCES

Bastion Y, Reyes F, Bosly A, Gisselbrecht C, Yver A, Gilles E, Maral J and Coiffier B (1994) Possible toxicity with the association of G-CSF and bleomycin. *Lancet* **343**: 1221–1222

Dirix LY, Schrijvers D, Druwe P, Van Den Brande J, Verhoeven D and Van Ooosterm AT (1994) Pulmonary toxicity and bleomycin. *Lancet* **344**: 56

Idell S, Kucich U, Fein A, Kueppers F, James HL, Walsh PN, Weinbaum G, Colmnn RW and Cohen AB (1985) Neutrophil elastase-releasing factors in bronchoalveolar lavage from patients with adult respiratory distress syndrome. *Am Rev Respir Dis* **132**: 1098–1105

Iki S, Yoshinaga K, Ohbayashi Y and Urabe A (1993) Cytotoxic drug-induced pneumonia and possible augmentation by G-CSF-clinical attention (letter). *Ann Hematol* **66**: 217–218

Katoh M, Shikoshi K, Takada M, Umeda M, Tsukahara T, Kitagawa S and Shirai T (1993) Development of interstitial pneumonitis during treatment with granulocyte colony-stimulating factor. *Ann Hematol* **67**: 201–202

Laver J and Moore MAS (1989) Clinical use of recombinant human hematopoietic growth factors. *J Natl Cancer Inst* **81**: 1370–1382

Lei Kik, Leung WT and Johnson PJ (1994) Serious pulmonary complications in patients receiving recombinant granulocyte colony-stimulating factor during BACOP chemotherapy for aggressive non-Hodgkin's lymphoma. *Br J Cancer* **70**: 1009–1013

Matthews JH (1993) Pulmonary toxicity of ABVD chemotherapy and G-CSF in Hodgkin's disease: possible synergy (letter). *Lancet* **342**: 988

Murayama J, Kawakami T and Togawa S (1994) Two cases of malignant lymphoma patient who developed interstitial pneumonia after CHOP-G therapy. *Med J Ibaraki Prefecture Hospital* **12**: 121–129

Niitsu N and Umeda M (1994) The effects of chemotherapy and G-CSF in patients with non-Hodgkin's lymphoma. *Chemotherapy* **42**: 346–350

Ohsaka A, Kitagawa S, Sakamoto S, Miura Y, Takanashi N, Takaku F and Saito M (1989) In vivo activation of human neutrophil functions by administration of recombinant human granulocyte colony-stimulating factor in patients with malignant lymphoma. *Blood* **74**: 2743–2748

© Cancer Research Campaign 1997

Ohsaka A, Saionji K, Sato N, Mori T, Ishimoto K and Inamatsu T (1993) Granulocyte colony-stimulating factor down-regulates the surface expression of the human leucocyte adhesion molecule-1 on human neutrophils *in vitro* and *in vivo*. *Br J Haematol* **84**: 574–580

Okubo Y and Nakazawa K (1993) Recombinant G-CSF and interstitial pneumonia during MACOP-B therapy in two cases of non-Hodgkin's lymphoma. *Jpn J Clin Hematol* **34**: 473–477

Pettengell R, Gurney H, Radford JA, Deakin DB, James R, Wilkinson PM, Kane K, Bentley J and Crowther D (1992) Granulocyte colony-stimulating factor to prevent dose-limiting neutropenia in non-Hodgkin's lymphoma: a randomized controlled trial. *Blood* **80**: 1430–1436

Sausville EA, Peisach J and Horwits SB (1978) Effect of chelating agents and metalions on the degradation of DNA by bleomycin. *Biochemistry* **17**: 2740–2746

Tazi A, Nioche S, Chastre J, Smiejan JM and Hance AJ (1991) Spontaneous release of granulocyte colony-stimulating factor (G-CSF) by alveolar macrophages in the course of bacterial pneumonia and sarcoidosis: endotoxin-dependent and endotoxin-independent G-CSF release by cells recovered by bronchoalveolar lavage. *Am J Respir Cell Biol* **4**: 140–147

Weiland Je, Davis WB, Holter JF, Mohanmed JR, Dorinsky PM and Gadek JE (1986) Lung neutrophils in the adult respiratory distress syndrome. Clinical and pathophysiologic significance. *Am Rev Respir Dis* **133**: 218–225

Wiley JS, Jamieson GP, Cebon JS, Woodruff RK, McKendric JJ, Szer J, Gibson J, Sheridan WP, Biggs JC and Rallings MC (1993) Cytokine priming of acute myeloid leukemia may produce a pulmonary syndrome when associated with a rapid increase in peripheral blood myeloblasts. *Blood* **82**: 3511–3512

© Cancer Research Campaign 1997